# Bacteria Are New Targets for Inhibitors of Human Farnesyltransferase

**DOI:** 10.3389/fmicb.2021.628283

**Published:** 2021-11-30

**Authors:** Lea Weber, Anna Hagemann, Jila Kaltenhäuser, Manuela Besser, Patrick Rockenfeller, Anja Ehrhardt, Ewa Stuermer, Hagen Sjard Bachmann

**Affiliations:** ^1^Centre for Biomedical Education and Research, Institute of Pharmacology and Toxicology, Witten/Herdecke University, Witten, Germany; ^2^Department of Translational Wound Research, Centre for Biomedical Education and Research, Witten/Herdecke University, Witten, Germany; ^3^Centre for Biomedical Education and Research, Institute of Biochemistry and Molecular Medicine, Witten/Herdecke University, Witten, Germany; ^4^Centre for Biomedical Education and Research, Institute of Virology and Microbiology, Witten/Herdecke University, Witten, Germany; ^5^Department of Vascular Medicine, University Heart Center, Translational Wound Research, University Medical Center Hamburg-Eppendorf, Hamburg, Germany

**Keywords:** farnesyltransferase, lonafarnib, tipifarnib, bacteria, antibiotics

## Abstract

Farnesyltransferase inhibitors (FTIs) are focus for the treatment of several diseases, particularly in the field of cancer therapy. Their potential, however, goes even further, as a number of studies have evaluated FTIs for the treatment of infectious diseases such as malaria, African sleeping sickness, leishmaniosis, and hepatitis D virus infection. Little is known about protein prenylation mechanisms in human pathogens. However, disruption of *IspA*, a gene encoding the geranyltranstransferase of *Staphylococcus aureus* (*S. aureus*) leads to reprogramming of cellular behavior as well as impaired growth and decreased resistance to cell wall-targeting antibiotics. We used an agar well diffusion assay and a time kill assay and determined the minimum inhibitory concentrations of the FTIs lonafarnib and tipifarnib. Additionally, we conducted cell viability assays. We aimed to characterize the effect of these FTIs on *S. aureus*, methicillin-resistant *Staphylococcus aureus* (MRSA), *Staphylococcus epidermidis* (*S. epidermidis*), *Escherichia coli* (*E. coli*), *Enterococcus faecium* (*E. faecium*), *Klebsiella pneumoniae* (*K. pneumoniae*), *Pseudomonas aeruginosa* (*P. aeruginosa*), and *Streptococcus pneumoniae* (*S. pneumoniae*). Both the FTIs lonafarnib and tipifarnib were capable of inhibiting the growth of the Gram-positive bacteria *S. aureus*, MRSA, *S. epidermidis*, and *S. pneumoniae*, whereas no effect was observed on Gram-negative bacteria. The analysis of the impact of lonafarnib and tipifarnib on common human pathogens might lead to novel insights into their defense mechanisms and therefore provide new therapeutic targets for antibiotic-resistant bacterial infections.

## Introduction

**FIGURE 1:**
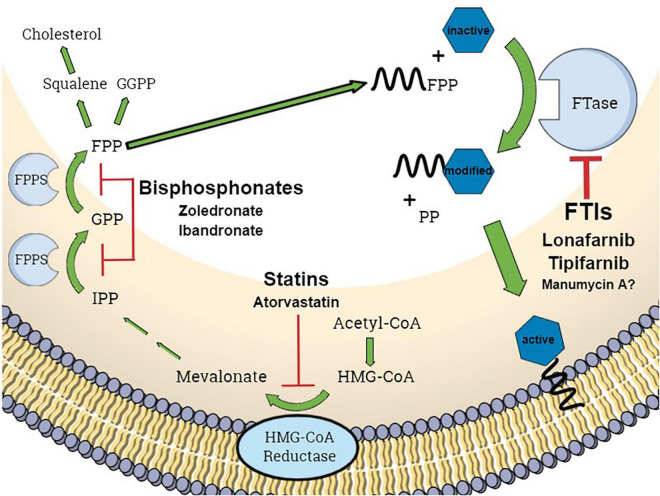
Schematic representation of the mevalonate pathway and protein farnesylation process in eukaryotic cells. GGPP, geranylgeranyl pyrophosphate; FPP, farnesyl pyrophosphate; FPPS, farnesyl pyrophosphate synthase; FTase, farnesyltransferase; FTIs, farnesyltransferase inhibitors; PP, pyrophosphate; IPP, isopentenyl pyrophosphate; blue hexagon, CAAX-motif protein. Graphic was created using *Mind the Graph*.

In eukaryotic cells, several proteins are converted into functional proteins by posttranslational modifications (PTMs) (Lane and Beese, 2006). These proteins are required for cell growth, differentiation, and apoptosis or general signaling pathway activity. One important PTM is prenylation, which is the covalent attachment of an isoprenoid residue to cysteine residues of proteins after translation. To date, more than 200 eukaryotic proteins are known to require prenylation modification (Wang and Casey, 2016). Isoprenoid residues are generated *via* the mevalonate pathway, a metabolic pathway by which squalene (and thus cholesterol) is synthesized from acetyl-CoA (Figure 1). The attachment of isoprenoid residues is catalyzed by different transferases: farnesyltransferase (FTase) and geranylgeranyltransferases I, II, and III (GGTaseI, GGTaseII, and GGTaseIII) (Jiang et al., 2018; Kuchay et al., 2019). FTase catalyzes the transfer of a farnesyl residue (a C15 isoprene unit) from farnesyl diphosphate [also called: farnesyl pyrophosphate (FPP)] to proteins with a CAAX C-terminal motif (Ochocki and Distefano, 2013), where C is a cysteine, the two A residues are aliphatic amino acids and X residue determines the specificity of the prenyltransferase (Figure 1). Prenylated proteins include members of the Ras, Rab, and Rho families; lamins; and the γ subunits of some G proteins (Lane and Beese, 2006). Hence, disruption of prenylation leads to the development of not only various pathologies of genetic origin, e.g., cancer, but also to osteoporosis and precocious aging (Gao et al., 2009). The use of farnesyltransferase inhibitors (FTIs) to disrupt the functionality of the oncogene Ras, e.g., appears to be a promising therapeutic approach. The two FTIs tipifarnib and lonafarnib (Norman, 2002; Equbal et al., 2008) play an important role in the therapy of some diseases, such as progeria and cancer as well as in pathogen-based diseases, such as hepatitis D (Lane and Beese, 2006; Capell et al., 2008; Koh et al., 2015; Caviglia and Rizzetto, 2020); however, the only approved FTI is lonafarnib, which was approved for the treatment of Hutchinson–Gilford progeria syndrome in 2020 (U.S. Food and Drug Administration, 2020).

The processes underlying prenylation by FTase or GGTases are well characterized in humans, however, little is known about similar pathways or orthologous proteins in bacteria. Maurer-Stroh et al. (2003) described the existence of protein prenyltransferases in vertebrates, nematodes, insects, plants, fungi, and protozoa. The conservation of these gene sequences in numerous species raises the question of whether these genes exist in bacteria and what their possible roles are. As far as we know, whether a farnesyltransferase exists in prokaryotes is largely unknown, and very few prokaryotic proteins contain a CAAX motif, which makes it difficult to establish the typical pattern of prenylation (Krute et al., 2015).

In *Streptomyces* species as well as in *Bacillus subtilis* prenyltransferases have been identified, and in *Bacillus* spp., the protein ComX, which is a quorum sensing pheromone, has been found to be prenylated at a tryptophan at the C-terminal end (Metzger et al., 2010; Bonitz et al., 2011; Novelli and D’Apice, 2012; Tsuji et al., 2012; Hirooka et al., 2020). In some other bacterial strains, the existence of prenyltransferases has been demonstrated, although these proteins play roles very different from those of eukaryotic farnesyltransferases, e.g., mediating the synthesis of ubiquinone (Marakasova et al., 2013). Thus, the identification of prenylated proteins and the underlying processes in bacteria could offer new opportunities to develop more targeted antibiotics. The antibiotic manumycin A was found to show antibacterial activity against Gram-positive bacteria such as *B. subtilis* (Zeeck et al., 1987a) and Gram-negative bacteria such as *Anaplasma phagocytophilum* (Xiong and Rikihisa, 2011). However, the mechanism responsible for the antibacterial effect remains elusive. Interestingly, this antibiotic is considered an FTI in eukaryotes (Hara et al., 1993).

For some human pathogens, such as *Escherichia coli* (*E. coli*), *Salmonella* spp., and *Legionella* spp., a hijacking of the host prenylation machinery has been postulated on the basis of modeling (Ivanov et al., 2010; Price et al., 2010). *Legionella* use the host prenylation machinery to mediate their membrane localization and association with specific organelles in the infected host (Ivanov et al., 2010). This phenomenon is exemplified by the injection of the effector protein ankyrin B (AnkB) by *Legionella pneumophila* into the host cell, where it undergoes farnesylation *via* the host prenylation machinery (Price et al., 2010). *Staphylococcus aureus* (*S. aureus*) employs the host cell prenylation machinery to promote its invasion: staphylococcal adhesins bind to the membrane of human cells by interacting with the CAAX-motif of Rac and CDC42 (Horn et al., 2008). Another study showed that inhibition of isoprenoid production in HUVECs by the HMG-CoA reductase inhibitor simvastatin prevents *S. aureus* invasion into host cells (Sattler et al., 1998).

The aim of our study was to investigate the effect of the FTIs tipifarnib and lonafarnib on several pathogenic Gram-positive and Gram-negative bacteria. The antibacterial effect of manumycin A on both Gram-positive and Gram-negative bacteria was shown; however, the underlying mechanisms remain elusive. Additionally, bioinformatic analyses were performed to determine the effects on a molecular level. *In silico* analyses were conducted to predict bacterial protein structures and motifs that share similarities with those of human prenyltransferases. The result of transcriptome analyses of FTI- and control-treated *S. aureus* are expected to shed light on transcriptional alterations indicating possible bacterial targets.

## Materials and Methods

### Chemicals

Lonafarnib and tipifarnib were purchased from SelleckChem (SelleckChem, Munich, Germany). DMSO was purchased from Sigma Aldrich (Sigma Aldrich, MO, United States).

### Bacterial Strains

The following bacterial strains were used: *S. aureus* (DSM-799, Leibniz-Institute DSMZ-German Collection of Microorganisms and Cell Cultures), methicillin-resistant *Staphylococcus aureus* (MRSA) (clinical isolate, kindly provided by B. Ghebremedhin, Helios University Medical Centre, Wuppertal, Germany), *Streptococcus pneumoniae* (*S. pneumoniae*) (kindly provided by A. Ehrhardt, Witten/Herdecke University, Witten, Germany), *E. coli* (DSM-11250, Leibniz-Institute DSMZ-German Collection of Microorganisms and Cell Cultures), *Pseudomonas aeruginosa* (*P. aeruginosa*) (DSM-939, Leibniz-Institute DSMZ-German Collection of Microorganisms and Cell Cultures), *Staphylococcus epidermidis* (*S. epidermidis*) (DSM-20044, Leibniz-Institute DSMZ-German Collection of Microorganisms and Cell Cultures), *Klebsiella pneumoniae* (*K. pneumoniae*) (DSM-30104, Leibniz-Institute DSMZ-German Collection of Microorganisms and Cell Cultures), and *Enterococcus faecium* (*E. faecium*) (DSM-2146, Leibniz-Institute DSMZ-German Collection of Microorganisms and Cell Cultures).

All bacterial strains except *S. pneumoniae* were cultured at 37°C in casein/soy peptone broth [CSB; 15 mg ml^–1^ casein peptone, 5 mg ml^–1^ sodium chloride, and 5 mg ml^–1^ soy peptone in A. *dest* (pH 7.2)], and on casein/soy peptone agar (CSA; 15 mg ml^–1^ agar in CSB, AppliChem, Darmstadt, Germany). *S. pneumoniae* was cultivated in lysogeny broth medium (LB; 10 mg ml^–1^ tryptone, 5 mg ml^–1^ yeast extract, 10 mg ml^–1^ sodium chloride in A. *dest*) for time kill assay and agar well diffusion tests and in M92-medium for the determination of the minimum inhibitory concentration (MIC).

The bacterial suspension was cultivated overnight at 37°C and shaken at 180 rpm under aerobic conditions. On the next day, bacterial suspensions were plated on agar plates and cultivated overnight at 37°C. After that, a single colony was picked for further cultivation.

### Agar Well Diffusion Test

Agar well diffusion tests were conducted in order to evaluate the antimicrobial effects on *S. aureus*, MRSA, *S. epidermidis*, *S. pneumoniae*, *E. coli*, *P. aeruginosa*, *K. pneumoniae*, and *E. faecium*. Agar plates were seeded with freshly prepared bacterial suspensions without a predefined OD_600_ to achieve a bacterial lawn. With the back of a sterile glass tip, three agar wells with a diameter of 0.3 cm were punched into the agar plates. The wells were loaded with 100 μl of tipifarnib (500 μM), lonafarnib (500 μM), or an equal amount of DMSO (1%) as control and the plates were incubated overnight at 37°C. The inhibition zone appearing around the wells was documented and analyzed using the *ImageJ* software.

### Determination of the Minimum Bactericidal Concentration and the Minimum Inhibitory Concentration

To determine the MIC of lonafarnib and tipifarnib against *S. aureus*, *S. pneumoniae*, *S. epidermidis*, and *E. coli*, the strains were grown overnight in M92-medium (*S. aureus* and *S. pneumoniae*) or CSB-medium (*E. coli* and *S. epidermidis*). The next day a freshly inoculated culture (0.1%) was grown for 4–5 h. The OD_600_ was determined and the culture was diluted to a 0.5 McFarland standard. The samples were inoculated with inhibitors diluted in 1% DMSO. A serial dilution of the inhibitors was made so that the concentration of DMSO was equal in every sample (1%). Bacteria treated with medium only were used as a negative control. Samples (100 μl) were inoculated into a 96 well plate and the optical density (OD_600_) was measured continuously for 24 h at 37°C with shaking at 600 rpm (SPECTROstar^Nano^, BMG Labtech). After that, 3 μl of the treated cultures was transferred to an agar plate and incubated overnight at 37°C. The minimal bactericidal concentration was determined as the lowest concentration that resulted in no visible bacterial growth (Mah, 2014).

### Time Kill Assay – Antibacterial Efficacy of Lonafarnib and Tipifarnib *in vitro*

For the analysis of the antibacterial effect of lonafarnib and tipifarnib we conducted a time kill assay on different bacterial strains. Bacterial suspensions were prepared in CSB and the suspension was adjusted *via* a spectrophotometer (EON Microplate Spectrophotometer, BioTek Instruments GmbH, VT, United States) to reach a 0.5 McFarland standard (∼1.5 × 10^8^ CFU/ml).

The inhibitors were added at concentration of 500 μM to the suspension and incubated for different time periods. The suspension was then serially diluted 10-fold in CSB, and 50 μl of each dilution was plated onto CSA plates. The plates were incubated at 37°C under aerobic conditions overnight. Colonies were counted *via* a colony-counter pen (Heathrow Scientific, Vernon Hills, IL, United States). All experiments were independently performed three times with triplicate samples in each experiment for test agents and duplicate samples for DMSO.

### Cell Culture

NIH3T3 cells (murine embryonal fibroblast cells) were provided by the Institute of Pharmacogenetics, University Hospital Essen, Essen, Germany. Cells were cultured in DMEM containing 10% FBS, 100 units/ml Pen/Strep (1%), and 500 μl gentamycin (50 mg/μl) in a humidified atmosphere at 37°C with 5% CO_2_. Cells were passaged every 3–4 days at a confluence of 90%.

### Cell Viability Assay (MTS)

For evaluation of cell viability, a CellTiter 96^®^ AQueous One Solution Cell Proliferation Assay (MTS) (Promega, WI, United States) was used. A total of 7,500 cells per well were seeded and grown in a monolayer. After 24 h, cells were incubated with lonafarnib and tipifarnib at different concentrations (3.6–500 μM) for 48 h. After incubation, the MTS assay was performed according to manufacturer’s protocol.

### Next Generation Sequencing

Next generation sequencing (NGS) analysis of *S. aureus* cells treated with both FTIs were conducted. Raw data were generated for *S. aureus* treated with lonafarnib (500 μM), tipifarnib (500 μM), and DMSO for 6 h. *S. aureus* was grown overnight in M92-medium and the next day, a freshly inoculated culture (0.1%) was grown for 4–5 h. The culture was diluted to a 0.5 McFarland standard. Samples were inoculated with lonafarnib (500 μM), tipifarnib (500 μM), or DMSO. After 6 h, cells were centrifuged and pellets were sent to Eurofins. RNA isolation and transcriptome analysis were performed according to the manufacturer’s protocols (Eurofins Genomics Germany GmbH, Ebersberg, Germany). In brief: RNA-Seq was performed on the Illumina sequencing platform (run type: paired end, read length: 2 × 150 bp, 30 Mio reads). Reads were aligned to the *S. aureus* genome and transcriptome. Table 1 lists the quality control statistics for the samples, and Table 2 shows the mapped read statistics.

**TABLE 1:** Quality control statistics for the samples.

Sample	Total reads	Discarded reads	Clean reads
Lonafarnib	82,483,146	1,787,796 (2.2%)	79,218,000 (96.0%)
Tipifarnib	80,289,784	1,939,198 (2.4%)	76,710,692 (95.5%)
Control	76,683,152	1,586,949 (2.1%)	73,790,208 (96.2%)

**TABLE 2:** Mapped read statistics determined for the samples.

Sample	QC passed reads	Mapped reads	% Mapped
Lonafarnib	79,218,000	61,222,779	77.28
Tipifarnib	76,710,692	55,908,112	72.88
Control	73,790,208	61,887,415	83.87

Eurofins provided a list of the differentially expressed genes. The unit *fragments per kilobase of exon per million fragments mapped* (FPKM) indicates the transcript abundance. *N* = 1 transcriptome for control, lonafarnib and tipifarnib.

### *In silico* Sequence Analyses

String analyses were conducted using the String database^1^ to find sequences homologous to the proteins ‘‘FNTA,’’ ‘‘FNTB,’’ and ‘‘PGGT1B.’’ Sequences with a coverage of 60% were considered similar. Furthermore, we used BLASTp (Protein Basic Local Alignment Search Tool) to identify similar sequences in bacteria.^2^ Analyses were performed *via* BLASTp (NCBI) searches with the annotated human FNTA and FNTB protein sequences as queries against the Bacteria (Bacteria taxid: 2) database. Hits for recognized proteins were counted when the sequences produced significant alignment (Query cover: 16–64%, *E* value: 3e−16 to 2.9, no cut-off).

A phylogenetic tree was constructed to visualize the homologs of FNTB in different bacterial strains.

### Statistics

For statistical analysis, GraphPad Prism 8 (GraphPad Software, Inc., La Jolla, CA, United States) was used. Data were evaluated by one-way ANOVA. Correction for multiple comparisons was performed using Tukey’s *post hoc* test.

The data are represented as the mean ± SD (standard deviation) from at least three independent experiments, unless otherwise stated. Statistical significance is indicated as follows: *p < 0.05; ^**^p < 0.01; and ^***^p < 0.001.

## Results

**FIGURE 2:**
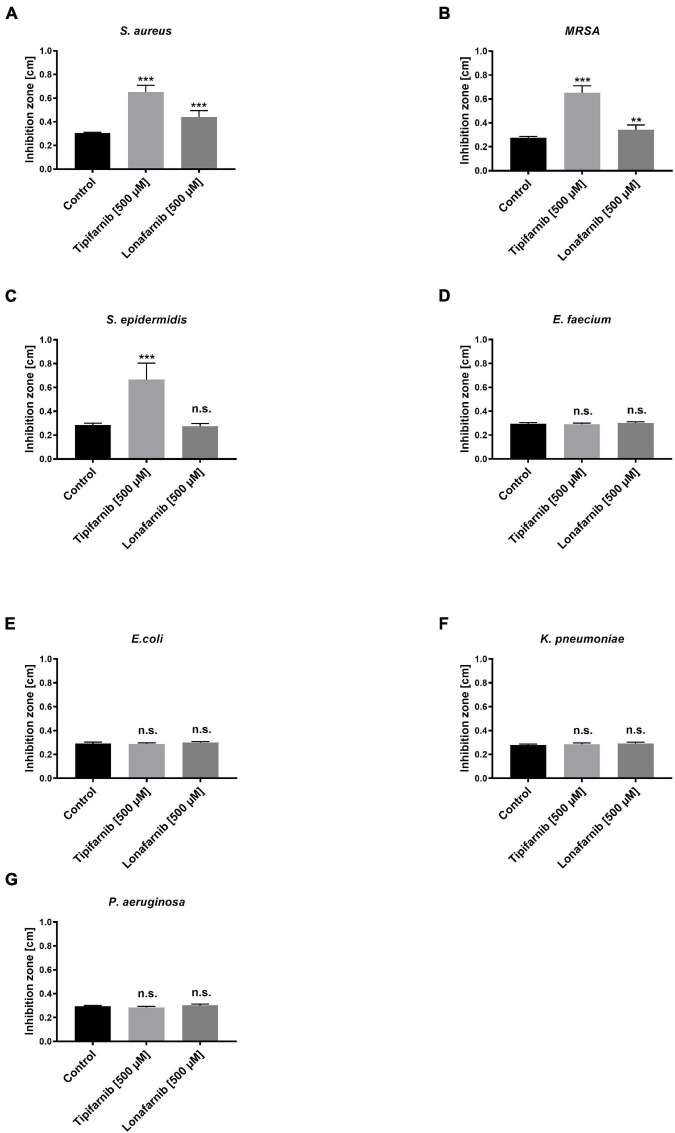
The agar well diffusion assay showed the effect of lonafarnib and tipifarnib on the formation of inhibition zones in cultures of different bacterial strains. Control (DMSO), lonafarnib, and tipifarnib (500 μM) were applied to agar plates inoculated with different bacterial strains [**(A)** *S. aureus*, **(B)** MRSA, **(C)** *S. epidermidis*, **(D)** *E. faecium*, **(E)** *E. coli*, **(F)** *K. pneumoniae*, and **(G)** *P. aeruginosa*]. Statistical significance was indicated as follows: **p < 0.01 and ***p < 0.001. Significant differences between lonafarnib- and tipifarnib-treated *S. aureus*, MRSA, and *S. epidermidis* is indicated as follows: ***. n.s. = not significant.

To elucidate the effect of the FTIs lonafarnib and tipifarnib on different bacterial strains, we initially conducted agar well diffusion assays with stationary/sessile bacteria. The inhibition zones were measured after treatment of different bacterial strains with lonafarnib and tipifarnib (Figure 2).

Treatment with lonafarnib and tipifarnib (500 μM) led to the formation of inhibition zones with average diameters of 0.44 and 0.65 cm, respectively, in *S. aureus* cultures. In MRSA cultures, lonafarnib and tipifarnib treatment resulted in the formation of significantly larger inhibition zones (0.34 and 0.65 cm; *p*-values: 0.0014 and <0.0001), than did DMSO treatment. Tipifarnib had a similar effect on *S. epidermidis*, resulting in inhibition zones with an average diameter of 0.67 cm (*p*-value < 0.0001). Lonafarnib had no significant effect (Figure 2). *E. coli*, *E. faecium*, *K. pneumoniae*, and *P. aeruginosa* were not affected by lonafarnib or tipifarnib in the agar well diffusion test, showing inhibition zones with average diameters of 0.28–0.30 cm (Figure 2). Due to the need for culture on blood agar plates, analysis of the inhibition zone of *S. pneumoniae* was impossible, as regardless of the product applied, the edges of the punched holes were discolored, possibly because of hemolytic processes. Because the agar well diffusion test revealed significant but small effects of treatment with lonafarnib and tipifarnib on *S. aureus*, MRSA, and *S. epidermidis* and the effects were not evaluable for *S. pneumoniae*, we aimed to further characterize the possible effect in a planktonic environment.

Additionally, we determined the MIC of lonafarnib and tipifarnib against *S. pneumoniae*, *S. aureus*, *S. epidermidis*, and *E. coli* over a time period of 24 h. Here, the MIC of lonafarnib was 12.5 μM against *S. pneumoniae*, 25 μM against *S. aureus*, and not evaluable against *S. epidermidis* due to the formation of precipitates (Table 3). Tipifarnib showed an MIC of 125 μM against *S. pneumoniae*, 500 μM against *S. aureus*, and 62.5 μM against *S. epidermidis* (Table 3). Similar to lonafarnib, tipifarnib occasionally precipitated at concentrations >125 μM. No inhibitory effect was observed against *E. coli*. The minimum bactericidal concentration (MBC) of tipifarnib was 2,500 μM against *S. aureus* and 500 μM against *S. epidermidis*. The MBC of lonafarnib was 32.5 μM against *S. aureus* and was not evaluable against *S. epidermidis*.

**TABLE 3:** Minimum inhibitory concentration (MIC) and minimum bactericidal concentration (MBC) of lonafarnib and tipifarnib on *S. aureus*, *S. epidermidis*, *S. pneumoniae*, and *E. coli*.

	MIC (μM)		MBC (μM)	
	Lonafarnib	Tipifarnib	Lonafarnib	Tipifarnib
*S. aureus*	25	500	32.5	2,500
*S. epidermidis*	N.d.	62.5	N.d.	500
*S. pneumoniae*	12.5	125	N/A	N/A
*E. coli*	No inhibition	No inhibition	N/A	N/A

*OD_600_ was measured for both strains treated with increasing concentrations of lonafarnib and tipifarnib. Concentrations used for MIC ranged from 0.78 to 100 μM (lonafarnib) and 3.6 to 500 μM (tipifarnib). Concentrations used for MBC ranged from 3.6 μM to 2.5 mM (tipifarnib) and 3.6 μM to 1 mM (lonafarnib). N.d., not determined, due to precipitations of the inhibitors the data are not evaluable.*

**FIGURE 3:**
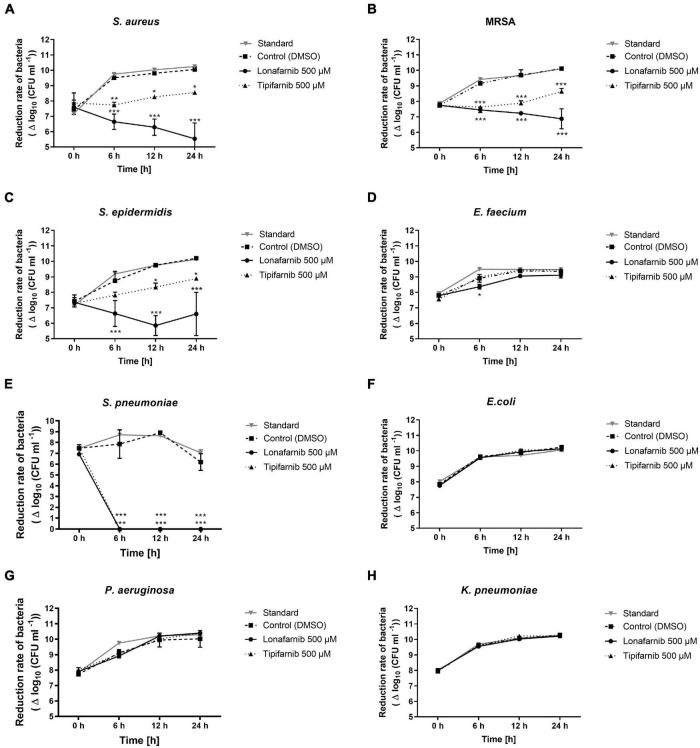
The time kill assay revealed the reduction rate of different bacterial strains after treatment with lonafarnib (500 μM) and tipifarnib (500 μM). The effect on **(A)** *S. aureus*, **(B)** *MRSA*, **(C)** *S. epidermidis*, **(D)** *E. faecium*, **(E)** *S. pneumoniae*, **(F)** *E. coli*, **(G)** *P. aeruginosa*, and **(H)** *K. pneumoniae* was evaluated. Statistical significance was indicated as follows: *p < 0.05; **p < 0.01; and ***p < 0.001.

After that, we conducted a modified time kill assay. Here, both FTIs induced a significant reduction after 6, 12, and 24 h (Figure 3). After 6 h, *S. pneumoniae* was completely eradicated upon treatment with both agents. Lonafarnib treatment led to a significant bacterial load reduction in the *S. aureus*, MRSA, and *S. epidermidis* cultures (Figure 3). In the *S. aureus* culture, lonafarnib treatment led to a persistent reduction in the bacterial count over a period of 24 h (5.54 ± 1.03 vs. 10.10 ± 0.20) (*p*-value < 0.0001). This effect was more pronounced in the MRSA culture, where treatment with lonafarnib reduced the bacterial count significantly to 6.87 ± 0.65 in comparison to that in the control culture (10.12 ± 0.07) (*p*-value < 0.0001). In the *S. epidermidis* culture, a significant reduction of up to 4 log units was observed (6.60 ± 1.40 vs. 10.20 ± 0.06) (*p*-value < 0.0001) after 24 h (Figure 3).

*Escherichia coli*, *K. pneumoniae*, and *P. aeruginosa* did not show any reductions in the bacterial load, although *E. faecium* showed a significant reduction after 6 h (8.40 ± 0.16 vs. 8.90 ± 0.25) and a slightly reduced but statistically non-significant reduction 24 h after lonafarnib treatment (9.11 ± 0.18 vs. 9.36 ± 0.08) (Figure 3).

Tipifarnib treatment initially decreased the bacterial count significantly in the *S. aureus* and MRSA cultures after 6 h (*p*-values: 0.0026 and 0.0001); however, the bacterial load recovered time-dependently over a period of 24 h (Figure 3). After 6 h, the bacterial load was reduced in the *S. aureus* culture compared to the DMSO-treated culture (7.74 ± 0.18 vs. 9.52 ± 0.32). Although the bacterial count subsequently increased afterward (8.55 ± 0.10 vs. 10.10 ± 0.20), it was still significantly lower than in the control culture. Similar results were obtained 24 h after tipifarnib treatment of MRSA (8.64 ± 0.19 vs. 10.12 ± 0.07). After 6 h of treatment, this effect was less pronounced in the *S. epidermidis* culture (7.82 ± 0.20 vs. 8.75 ± 0.61); however, the trend was the same. No bacterial load reduction was observed for *E. coli*, *E. faecium*, *K. pneumoniae*, or *P. aeruginosa* with tipifarnib (Figure 3).

**FIGURE 4:**
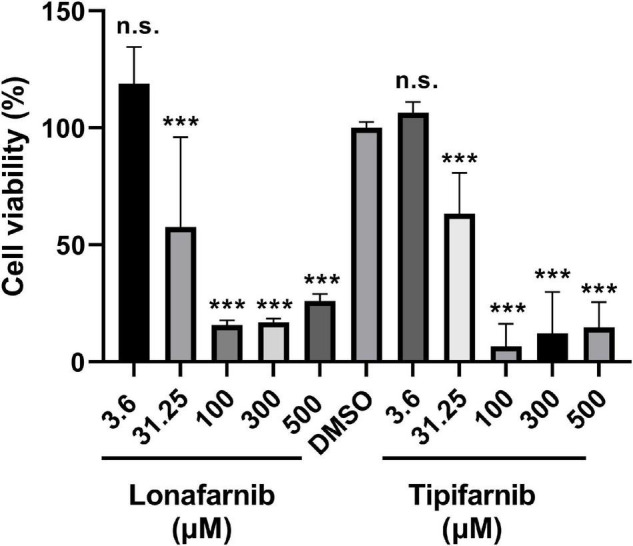
Cell viability assay in NIH3T3 cells after treatment with lonafarnib and tipifarnib. Cells were incubated with the FTIs at different concentrations for 48 h. Statistical significance is indicated as follows: ***p < 0.001. n.s. = not significant.

Additionally, we tested the effect of the FTIs using comparable concentrations in eukaryotic cells. Therefore, cell viability assays were conducted using NIH3T3 cells treated with lonafarnib and tipifarnib for 48 h (Figure 4). Lonafarnib treatment resulted in reductions in cell viability of up to 85 and 74% (*p*-values < 0.0001) at high concentrations (100 and 500 μM, respectively) and a moderate reduction in cell viability (43%) at a low concentration (31.25 μM, *p*-value < 0.0001). Similar results were obtained for tipifarnib treatment. Tipifarnib treatment led to a reduction in cell viability of 81% at a concentration of 100 μM and a moderate reduction (37%) at a concentration of 31.25 μM (both *p*-values < 0.0001) (Figure 4).

**FIGURE 5:**
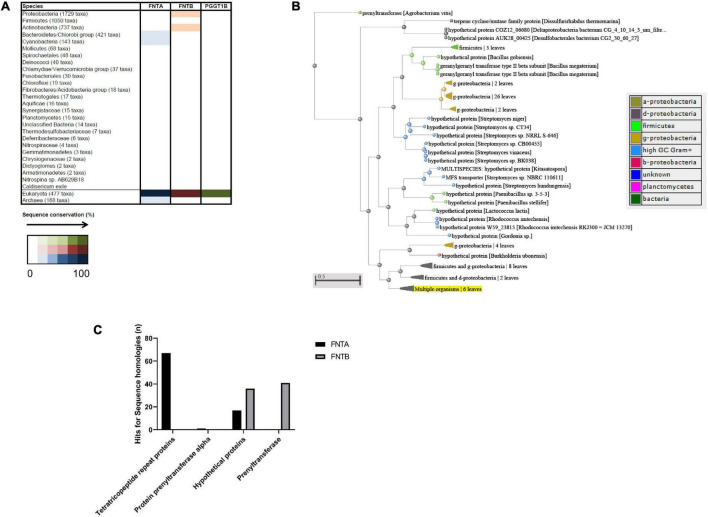
*In silico* sequence analysis of FNTA and FNTB in bacteria. **(A)** String analysis of the three proteins “FNTA,” “FNTB,” and “PGGT1B.” The color scheme indicates the number of homologous sequences in the respective taxon. **(B)** BLASTp analysis of multiple sequences from FNTA and FNTB in bacteria. Blue bar: FNTA; red bar: FNTB. **(C)** Representative phylogenetic tree of FNTB homologs in different bacterial strains. The color of the nodes represents the different classes of bacteria, shown in the table. The scale bar indicates the evolutionary distance between the species.

To identify possible bacterial targets, we conducted *in silico* analyses and searched for homologous sequences among bacterial taxons (Figure 5A). BLASTp (NCBI) searches against bacteria were conducted with the annotated human FNTA and FNTB protein sequences as query sequences. FNTA showed 67 hits for homologous sequences for “tetratricopeptide repeat proteins,” one for “protein prenyltransferase alpha,” 17 for a “hypothetical protein,” and none for “prenyltransferase” (Figure 5C). FNTB revealed sequence homology hits for “hypothetical protein” (36) and “prenyltransferase” (41) (Figure 5C). Therefore, these analyses revealed a number of homologous proteins, indicating putative targets of FTIs. The phylogenetic tree shows these data together with the corresponding species and their relationships (Figure 5B). In the interest of clarity, some leaves are summarized, e.g., those for the “g-proteobacteria.” The branch “Multiple organisms” highlighted in yellow represents the sequence of the initial query “FNTB” (Figure 5B).

**FIGURE 6:**
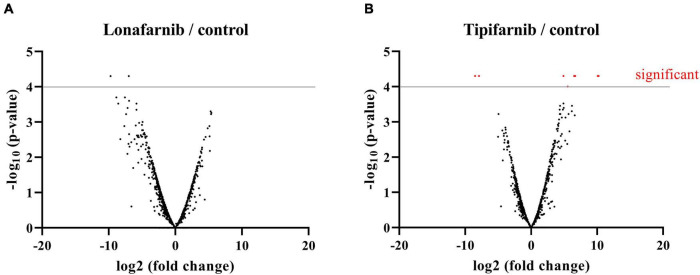
Volcano plots visualizing NGS data of *S. aureus* treated with lonafarnib (500 μM), tipifarnib (500 μM), or DMSO for 6 h. Analysis revealed differentially expressed genes. **(A)** Differentially expressed genes in *S. aureus* upon treatment with lonafarnib. **(B)** Differentially expressed genes in *S. aureus* upon treatment with tipifarnib.

Additionally, Next Generation Sequencing analysis was performed. For this analysis, *S. aureus* was treated with lonafarnib (500 μM), tipifarnib (500 μM), or DMSO for 6 h (Figure 6).

The treatment of *S. aureus* with tipifarnib induced significant transcriptional changes. Significant downregulated genes are: *Hld*, encoding delta-hemolysin (SAOUHSC_02260), *SceD*, encoding a transglycosylase (SAOUHSC_02333), *SsaA*, encoding the staphylococcal secretory antigen (SAOUHSC_02576), *betA*, encoding an oxygen-dependent choline dehydrogenase (SAOUHSC_02932), a gene encoding a multiple sugar-binding transport ATP-binding protein (SAOUHSC_00175), a multidrug ABC transporter permease (SAOUHSC_01312), and two hypothetical proteins (SAOUHSC_02640, SAOUHSC_A00526) (Figure 6). The significant upregulated genes are: SAOUHSC_01311, encoding an ABC-transporter, *HrtB*, encoding a hemin transport system permease protein (SAOUHSC_02641), and *betB*, encoding the betaine aldehyde dehydrogenase (SAOUHSC_02933) (Figure 6). The treatment of *S. aureus* with lonafarnib induced no significant transcriptional changes (Figure 6).

## Discussion

In this study, we investigated the effect of the FTIs lonafarnib and tipifarnib on different prokaryotes for the first time. In eukaryotes, farnesylation plays a major role in many diseases and its inhibition by FTIs is under investigation for the treatment of diseases such as cancer, progeria, and hepatitis delta (Gao et al., 2009; Koh et al., 2015; Gordon et al., 2018; Waller et al., 2019).

Five Gram-positive and three Gram-negative bacteria were analyzed to determine their sensitivity to the FTIs lonafarnib and tipifarnib. Here, inhibition zone tests revealed a bactericidal effect of tipifarnib on *S. aureus*, *S. epidermidis*, and MRSA, as well as a bactericidal effect of lonafarnib on *S. aureus* and MRSA. The results of time kill assays were similar; however, lonafarnib showed significantly stronger bacterial load reductions than tipifarnib in the *S. aureus*, MRSA, *S. epidermidis*, and *S. pneumoniae* cultures. Tipifarnib showed similar trends, but seemed to be less potent. This apparent discrepancy with the results of the agar well diffusion assay might be due to the different diffusion potentials resulting from the molecular structures of the inhibitors. The higher molecular weight of lonafarnib might influence its equal effective diffusion through agar, as observed for tipifarnib (Bauer et al., 1966; Le et al., 2020). None of the tests revealed any effects of lonafarnib or tipifarnib on Gram-negative bacteria, possibly due to the inability of these FTIs to pass through the bacterial membranes and wall. This problem will be addressed by overcoming the membrane barrier using electroporation in future studies.

Analysis of the MBC and MIC revealed concentrations in the low two-digit micromolar range for lonafarnib and in the low three-digit micromolar range for tipifarnib, depending on the bacterial strain. Therefore, we analyzed the viability of eukaryotic cells under treatment with these FTIs at set concentrations. Both FTIs had a significant and concentration-dependent impact on cell viability at concentrations in the two-digit micromolar range, resulting in reductions in viability of up to 75%. These experiments showed that the concentrations used are capable of evoking specific effects, which might suggest a specific effect in bacteria as well. However, these experiments also showed that these two FTIs are presumably not suitable for antibiotic therapy due to the reduction in the viability of eukaryotic cells. Kaplancikli et al. (2012) evaluated the *in vitro* cytotoxicity of new antimicrobial agents, carbazole derivatives, and found that they exerted antimicrobial effects in NIH3T3 cells in an MTS assay. Here, the IC50 ranged from 11 to 300 μg/ml, depending on the compound. When the cells were treated with 49 μg/ml (100 μM) tipifarnib and 63.8 μg/ml (100 μM) lonafarnib, we observed reductions in cell viability of up to 75% (lonafarnib) and 90% (tipifarnib). This result indicates that our FTI exhibit cytotoxicity at the same concentration range as other antimicrobial agents; however, a reduction in cytotoxicity due to molecular modifications is preferable (Kaplancikli et al., 2012). Thus, lonafarnib and tipifarnib could be lead compounds for the development of new antibiotic therapies. This kind of approach is not unusual and was used for the successful synthesis of the compound PK150 from sorafenib. Sorafenib, an anticancer drug with weak antibacterial activity was used as the lead compound for the development of PK150, which is 10 times as effective as sorafenib and even capable of eradicating MRSA without targeting human kinases (Le et al., 2020).

The molecular mechanisms underlying the observed FTI-induced effects in bacteria remain elusive. The existence of prenyltransferases has been described in vertebrates, insects, nematodes, fungi, protozoa, and plants, however, only little is known about these proteins in bacteria (Maurer-Stroh et al., 2003).

Krute et al. (2015) generated a *S. aureus* mutant lacking *IspA*, coding for a geranyltranstransferase that synthesizes FPP in *S. aureus* [in eukaryotes, farnesyl pyrophosphate synthase (FPPS) is the protein with the corresponding function; Figure 1]. This mutation showed several pleiotropic effects, such as a complete lack of pigmentation and an ability to form only small colonies. From a therapeutic point of view, this mutation has desirable effects, such as impairing growth and increasing sensitivity to antibiotic substances (Krute et al., 2015). Similarly, another FTI, that is also a non-specific inhibitor of several other eukaryotic targets, i.e., the antibiotic manumycin A, has been found to be effective against fungi and bacteria (Zeeck et al., 1987b; Qiao et al., 2017). Other enzymes involved in isoprenoid metabolism in pathogens have also been successfully targeted by drugs already in clinical use: Schmidberger et al. (2015) revealed the existence of a FPPS (FPP) in *P. aeruginosa*, that is inhibited by ibandronate and zoledronate, two substances used as inhibitors of the human homolog.

In addition, the necessity of protein prenylation has been shown for several pathogens that manage to hijack human prenyltransferases for that purpose, e.g., *Salmonella typhimurium* and *L. pneumophila*. The latter uses the host prenylation machinery to prenylate AnkB, which allows the bacteria to infect alveolar macrophages (Marakasova et al., 2013; Michard and Doublet, 2015). Furthermore, geranylgeranylation of SifA by the host organism regulates the formation of tubular membranous structures that are needed for the survival and infection process of *S. typhimurium* (Reinicke et al., 2005; Marakasova et al., 2013). *S. aureus* might also use the host cell prenylation machinery for its invasion, beginning with binding of staphylococcal adhesins to human extracellular matrix proteins to induce its internalization (Horn et al., 2008). Simvastatin is thought to suppress this actin turnover used for streptococcal entry, most likely *via* suppression of HMG-CoA reductase, leading to a reduction in the biosynthesis of mevalonate and different intermediates, such as farnesylpyrophosphate (Horn et al., 2008). These findings show that prenylation in prokaryotes plays a decisive role in different processes, indicating promising targets for antipathogenic therapies.

The dependence of these pathogens on human prenyltransferases does not exclude the existence of bacterial prenyltransferases. Even the first autotrophic organisms, the cyanobacteria, seemed to use a geranyltransferase for the biosynthesis of piricyclamide (Morita et al., 2018). The antibiotic-producing bacterium *Streptomyces* uses its prenyltransferase for the synthesis of aromatic compounds such as lipoquinones (Marakasova et al., 2013). *S. coelicolor A3* and *S. niveus* can express two prenyltransferases, SCO7190 and NovQ, which prenylate 1,6-DHN, 2,7-DHN and naringenin (SCO7190), and phenylpropanoids (NovQ).

We aimed to investigate sequential homologous structures between bacteria and the subunits of eukaryotic CAAX-prenyltransferases in all bacteria by *in silico* proteome analyses. Here, several hits for different sequence alignments between farnesyltransferase subunits α (FNTA) and β (FNTB) were detected and suggest the existence of possible FTI targets in bacteria. Strikingly, the hits were allocated to both Gram-positive and Gram-negative bacteria. However, the presence of proteins with sequence homology could suggest the existence of similar proteins in bacteria and has possible evolutionary significance, but does not sufficiently prove the existence or functionality of such proteins.

Next, we aimed to specify the FTI-induced effects on the transcriptome of one of the bacterial strains impaired by FTI treatment, *S. aureus*. Therefore, we conducted Next generation sequencing analyses of FTI-treated *S. aureus* cells and identified several transcriptional alterations. The treatment of *S. aureus* by tipifarnib led to several changes in the bacterial transcriptome, especially in several transport proteins. The upregulation of SAOUHSC_01311, a gene related to membrane transport, is in accordance with observations of the transcriptional changes of *S. aureus* upon epigallocatechin gallate, a polyphenol displaying strong antibacterial activity against Gram-positive bacteria (Kitichalermkiat et al., 2020).

Furthermore, the treatment of *S. aureus* by tipifarnib led to the upregulation of *HrtB*, part of a hemin ABC transporter permease, which protects *S. aureus* against hemin toxicity (Attia et al., 2010). This might be a reaction to the rising concentration of tipifarnib in the cell.

Strikingly, *Hld*, which encodes delta-hemolysin is significantly downregulated upon tipifarnib treatment. Delta-hemolysin plays a major role in pathogenesis of several diseases caused by *S. aureus* and helps to lyse the membrane of the host cells in order to evade the immune system (Divyakolu et al., 2019). The inhibition of the hemolysin synthesis and the downregulation of the *agr* system, that controls delta hemolysin as well as the expression of virulence factors, is a high goal in the development of anti-virulence drugs (Divyakolu et al., 2019). Overall, our results suggest the afore-mentioned genes as interesting candidates for further studies to identify the underlying molecular pathways and FTI targets. A promising approach might be the characterization of FTI effects by related *S. aureus* deletion mutants and screening of respective libraries (Fey et al., 2013).

The treatment of *S. aureus* with lonafarnib did not result in significant changes in the transcriptome; however, maybe there are changes after longer incubation times.

**FIGURE 7:**
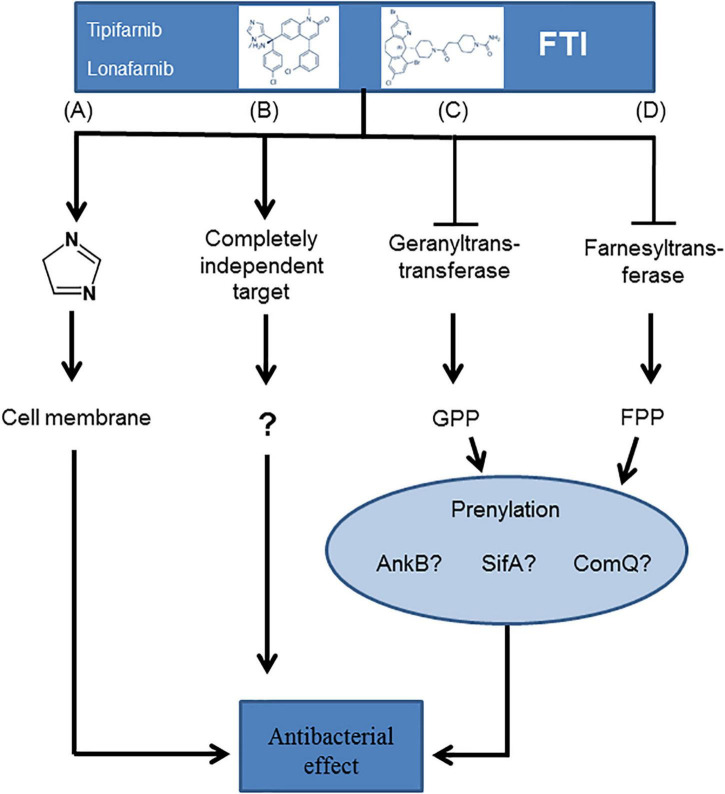
Hypotheses of the potential molecular basis for the growth inhibition of Gram-positive bacteria upon FTI treatment. The flow chart contrasts the different approaches: **(A)** imidazole-based antibacterial mechanisms, **(B)** completely unknown targets, **(C)** an effect based on inhibition of a geranyltranstransferase, or **(D)** a farnesyltransferase resulting in a shift in the prenylation type.

In summary, we were able to demonstrate the antibacterial potency of the FTIs lonafarnib and tipifarnib against Gram-positive bacteria. We developed different hypotheses elucidating the molecular basis of these bactericidal effects (Figure 7). These antibacterial effects could be due to the chemical structure, which is somewhat similar to that of imidazole, a known antibiotic substance (Rani et al., 2013; Figure 7A). Indeed, FTIs might also have completely independent and yet unknown targets (Figure 7B). Other possible mechanisms are inhibition of a geranyltranstransferase (Figure 7C) or a farnesyltransferase (Figure 7D), resulting in the lack of prenylation of diverse proteins, e.g., AnkB, SifA, or ComQ.

## Conclusion

In summary, this basic research investigated the effect of FTIs on common bacterial strains. Both tested FTIs, lonafarnib and tipifarnib, were effective against some Gram-positive bacteria at concentrations suggesting specific effects. New substances with yet unknown targets in bacteria could help to overcome the development of resistance, as the increasing bacterial resistance to common antibiotics is a growing problem. Due to their cytotoxicity at higher concentrations, lonafarnib and tipifarnib need to be considered as lead compounds for further investigation. Proteome and transcriptome analyses revealed possible bacterial targets responsible for the FTI-induced effects on *S. aureus*.

## Data Availability Statement

For string analyses data from FNTA, FNTB, and PGGT1B were taken from BRENDA (www.brenda-enzymes.org) using the following numbers: BRENDA:EC2.5.1.58 and BRENDA:EC2.5.1.59 and STRING-DB was used for analyzing the data (https://string-db.org/). For BLASTp analysis, sequence data were taken from NCBI (https://www.ncbi.nlm.nih.gov/) and analyses were conducted using the Basic Local Alignment Search Tool (https://blast.ncbi.nlm.nih.gov/Blast.cgi). For protein farnesyltransferase/geranylgeranyltransferase type-1 subunit alpha, the NCBI Reference Sequence is: NP_002018.1. For protein farnesyltransferase subunit beta the NCBI Reference Sequence is: NP_002019.1.

## Author Contributions

HB: project conceptualization. LW, AH, JK, and PR: methodology. LW, AH, MB, and JK: formal analysis and investigation. LW, ES, and HB: manuscript writing. HB, AH, and AE: manuscript review and editing. LW and AH: data visualization. HB and ES: supervision. All authors contributed to the article and approved the submitted version.

## Conflict of Interest

The authors declare that the research was conducted in the absence of any commercial or financial relationships that could be construed as a potential conflict of interest.

## Publisher’s Note

All claims expressed in this article are solely those of the authors and do not necessarily represent those of their affiliated organizations, or those of the publisher, the editors and the reviewers. Any product that may be evaluated in this article, or claim that may be made by its manufacturer, is not guaranteed or endorsed by the publisher.

## Footnotes

1
https://string-db.org/

2
https://blast.ncbi.nlm.nih.gov/Blast.cgi

## Abbreviations

DMSO: dimethyl sulfoxide
FTI: farnesyltransferase inhibitor
MRSA: methicillin-resistant *Staphylococcus aureus*
CSB: casein/soy peptone broth
CSA: casein/soy peptone agar.
